# The impact of urban low-carbon incentive policy on enterprise transformation and upgrading: Evidence from a quasi-natural experiment in China

**DOI:** 10.1371/journal.pone.0335621

**Published:** 2025-10-29

**Authors:** Yuanrui He, Mingzeng Yang

**Affiliations:** School of Accountancy, Shandong University of Finance and Economics, Jinan, Shandong, China; Xi'an University of Architecture and Technology, CHINA

## Abstract

As carbon emissions in China continue to rise and the cost advantage in the global value chain diminishes, enterprise transformation and upgrading has emerged as a new engine for economic growth. By implementing low carbon incentive policies, the government aims to spur corporate self‑innovation and phase out obsolete capacity, thereby boosting resource use efficiency and curbing environmental pollution. This paper examines the impact of China’s low carbon incentive policies on enterprise transformation and upgrading, with a particular focus on the role and mechanisms of urban environmental policy in this process. Employing a multi-period difference in differences approach, we analyze how the low carbon city policy affects the transformation and upgrading of Chinese listed firms. The results show that the low carbon city policy significantly enhances enterprise transformation and upgrading at the 1% level: participation in the low carbon city policy raises the composite index of enterprise transformation and upgrading by 0.012. We further explore the moderating role of enterprise green development level by incorporating it into our model of low carbon city policy effects. The findings reveal that firms exhibiting higher green total factor productivity, as well as those adopting green innovation and green management practices, display stronger adaptability to the low carbon city policy. Finally, both heterogeneity and dynamic analyses indicate that, over the medium to long term, the low carbon city policy continues to promote enterprise transformation and upgrading. In sum, the low carbon city policy not only provides exogenous momentum for enterprise transformation and upgrading but also interacts synergistically with firms’ green development to guide them toward more efficient and sustainable transformation and upgrading.

## 1. Introduction

China’s economic growth has decelerated, and its low cost advantage in the global value chain has progressively weakened. The challenges arising from the deterioration of China’s ecological environment have constrained the continuation of an extensive economic structure [1]. Therefore, seeking new growth requires the optimization and upgrading of the economic structure. At the micro level, a key factor in this process is the ability of enterprises to undergo transformation and upgrading. As a critical participant in the global value chain, China’s approach to optimizing its economic structure cannot simply be to eliminate low-end and highly polluting enterprises. In critical sectors like food and energy, China must continue to depend on its production capabilities to secure a stable supply of these essential resources. Due to geopolitical tensions and trade barriers, simply eliminating low-end enterprises can cause instability in the prices and supply of crucial products like energy and resources, threatening people’s livelihoods and national security. Therefore, it is crucial to help enterprises align with the trends of economic structural optimization and upgrading. This will enable them to achieve transformation and upgrading successfully. The notion of enterprise transformation and upgrading was first advanced by Gereffi (1999) [2]. However, a unified and explicit definition remains lacking [3]. In this study, enterprise transformation and upgrading are defined as a multidimensional, dynamically evolving developmental process whose core lies in enhancing firms’ market competitiveness through technology-driven innovation, improved resource allocation efficiency and value chain advancement [4–6]. Against the backdrop of macro level structural optimization and upgrading, supporting enterprises in realizing such transformation from a micro perspective is not only a pivotal strategy for bolstering corporate competitiveness. It is also a crucial practice for ensuring China’s sustainable development.

However, notwithstanding the macro level emphasis on the necessity of transformation and upgrading, firms are struggle to independently advance their own upgrading due to the dual constraints of low value chain positioning and insufficient transformational impetus. On the one hand, in the process of integration into global value chains, the “captivity effect” has emerged. This effect occurs when lead firms assume gatekeeping roles, leading Chinese enterprises to over rely on imported intermediate inputs for assembly and processing [7]. As a result, they confront persistently high energy consumption and low product value addition [8]. At the same time, they exhibit limited upstream value creation capacity and bearing the environmental costs associated with pollution displacement [9]. On the other hand, in international markets most Chinese firms occupy the supplier tiers of global value chains (i.e., GVC suppliers) and thus have little incentive to pursue innovation or develop new demand as a means of transformational upgrading [10]. Consequently, the high financial costs and significant risks associated with upgrading further diminish enterprises’ motivation to embark on such processes.

Within this context, the extant literature from Chinese firms’ lack of sufficient impetus for transformation and upgrading has systematically examined the factors influencing their transformation and upgrading. These factors are explored from both endogenous and exogenous driving force perspectives. At the endogenous level, technological capability, marketing capability and interaction with upstream value chain actors [11], full utilization of resources and skills [12], digital transformation [13], external learning mechanisms in networks [14], intra firm knowledge spillover [15], and entrepreneurial competence [16] are all important factors promoting enterprise upgrading. At the exogenous level, market demand in developed economies and the import of high-quality intermediate products [17], blockchain technology [4], energy infrastructure [18], and the business environment ecosystem [19] likewise drive upgrading. Policy instruments further constitute a vital exogenous impetus: vulnerability shocks induced by national policy can trigger enterprise transformation and upgrading [10]; robust government regulation and legislation can achieve process and product upgrading [20]; and environmental regulation policies can similarly promote upgrading [5,21]. For example, China’s green credit policy and its implementation of cleaner production standards have both facilitated enterprise transformation and upgrading [22,23]. However, few studies have integrated LCCP with enterprise transformation and upgrading. Given LCCP’s role as a key urban environmental regulation measure, its potential as a critical exogenous driver of firm-level upgrading requires urgent both theoretical and empirical investigation.

Since its implementation, the LCCP has played a critical role in fostering synergistic gains in environmental protection and economic development. Since the proposal of the LCCP in China in 2010, three batches of LCCP have been implemented across 81 cities in 2010, 2012, and 2017. In the 2010, the “notice on launching low-carbon pilot provinces and cities”, it was stated that “the adjustment of industrial structure, optimization of energy structure, energy conservation and efficiency enhancement, and the expansion of carbon sinks should be integrated” [24]. The “notice on launching low-carbon pilot provinces and cities” states that “the adjustment of industrial structure, optimization of energy structure, energy conservation and efficiency enhancement, and the expansion of carbon sinks should be integrated.” This indicates that the original intent of the LCCP also encompassed driving enterprise transformation and upgrading. While environmental regulations may initially increase production costs and reduce enterprise competitiveness, they can trigger significant technological innovations, laying the foundation for long-term growth and sustainable development [25]. When carefully designed and effectively implemented environmental regulations can stimulate corporate innovation, offsetting the additional costs of compliance and creating a win-win outcome for environmental protection and business development [26]. The LCCP constitutes a suite of regulatory instruments under which municipalities voluntarily undertake low carbon initiatives [27]. In practice, it allows cities to adapt measures according to local conditions and promotes coordinated pollution control among municipalities [28]. Consequently, it helps cities to set environmental policy standards rationally and contributes to ensuring that the Porter effect of the LCCP outweighs its cost effect [29]. This, in turn, ultimately fostering simultaneous improvements in firms’ environmental and economic performance.

Within this context, the extant literature largely concurs that the LCCP exerts a positive influence on both firms’ economic and environmental performance. In terms of economic performance, the LCCP has been shown to spur technological innovation and reduce R&D manipulation [30]. Although the substantive costs associated with these innovations increase firms’ financial pressures, such pressures in turn compel strategic adjustments that enhance production efficiency and resource utilization [31]. Moreover, the LCCP induced technological innovation, improved production efficiency, optimized resource allocation, and upgraded product quality collectively contribute to gains in total factor productivity [32]. The LCCP also stimulates entrepreneurial activity [33], and facilitates green mergers and acquisitions [34]. In terms of environmental performance, the LCCP significantly promotes green innovation [35], and low-carbon innovation [36,37], thereby enhancing firms’ environmental outcomes [38]. It further drives reductions in carbon emissions [39,40], energy savings [41], and curtails environmental noncompliance [42]. Concurrently, the policy heightens executives’ environmental awareness [43], attracts green investors [44], improves ESG performance [45], and advances clean production and green transformation [28,46].

Based on the above research, it is evident that LCCP can effectively stimulate firms to engage in innovative activities, enhance production efficiency, optimize resource allocation, and adopt environmentally friendly practices. These key factors constitute the essential foundation and prerequisite for driving corporate transformation and upgrading. Moreover, one goal of LCCP is to promote industrial upgrading [47]. At the micro level, industrial upgrading is ultimately realized through firms’ technological innovation, improvements in energy efficiency, and adjustments to their products and organizational structures. LCCP raise emission costs and providing green credit and tax incentives. This policy exerts both “pressure” and “incentive” effects on firms, thereby accelerating their transformation. Therefore, there exists an inherent linkage between LCCP and corporate transformation. However, few studies have directly explored this intrinsic relationship.

Therefore, this paper focuses on the impact of urban environmental policies by analyzing whether LCCP affect corporate transformation and upgrading. It also explores the intrinsic mechanisms underlying their relationship. To draw lessons from diverse urban environments, the pilot cities span multiple regions, development stages, and resource endowments. Such variation ensures that, prior to policy rollout, pilot and non-pilot cities were statistically comparable. This provides a strong counterfactual for estimating policy effects and constituting a quasi-natural experimental framework for our analysis.

The present work makes three key contributions. First, unlike prior research that evaluates industrial upgrading only at the city level, we adopt a multi period difference-in-differences design at the firm level. This approach allows us to pinpoint heterogeneous firm responses, thereby enriching the literature on how LCCP drives corporate transformation. Second, we are the first to operationalize firms’ green development along three dimensions: GTFP, green innovation, and green management. Additionally, we integrate these metrics into the mechanism analysis of LCCP’s effects on firm upgrading. Finally, we introduce a heterogeneous, multi-period DID estimator to assess how firms’ transformation outcomes vary with the timing of city participation in the LCCP. We trace for the first time the dynamic evolution of LCCP’s impact on corporate transformation over time.

The rest of this paper is structured as follows: Section 2 provides a literature review; Section 3 outlines the research hypotheses regarding the effect of LCCP on enterprise transformation and upgrading; Section 4 details the data construction process and empirical model setup; Section 5 offers empirical tests and mechanism analysis; and Section 6 presents the conclusion of this paper.

## 2. Theoretical analysis and research hypotheses

### 2.1. LCCP and enterprise transformation and upgrading

According to the definition, the focus of enterprise transformation and upgrading is on innovation, improving the efficiency of resource allocation, and enhancing the value chain. Moreover, LCCP positively influences enterprise green innovation, resource‑allocation efficiency, and value‑chain enhancement [32,38,48,49].

First, LCCP can stimulate firm level innovation, thereby facilitating enterprise transformation and upgrading. On the one hand, during policy implementation, pilot cities have employed various command-and-control environmental regulation instruments, such as environmental laws and low-carbon entry thresholds, to compel firms to innovate. Specifically, pilot cities like Jinan and Guiyang have adopted pollution control and clean-energy development statutes to strictly limit greenhouse-gas emissions, while jurisdictions such as Shijiazhuang and Nanchang have enacted local regulations that mandate more stringent phase-out standards to accelerate industrial upgrading. These measures increase firms’ compliance costs, motivating them to recoup expenses through technological and process innovations. On the other hand, LCCP not only increases public investment in science and technology R&D, but also fosters the growth of market-based environmental regulation tools, such as green finance and trade-credit financing, to support corporate innovation [50–52]. By offering innovation grants and reducing financing costs, these instruments enable firms to mobilize greater financial resources for R&D activities. Since innovation is the cornerstone of enterprise transformation and upgrading [53,54]. Therefore, LCCP leverages both command-and-control and market-based environmental regulation tools to enhance firms’ innovative capacity, furnishing robust momentum and institutional safeguards for their structural transformation.

Secondly, LCCP can enhance firms’ resource allocation efficiency [32], thereby promoting their transformation and upgrading. On the one hand, municipal environmental regulations raise firms’ compliance costs for carbon emissions, curbing high input, high energy consumption development models and directly improving resource allocation efficiency. Under the pressure of LCCP, firms or sectors facing high carbon-control costs encounter greater survival challenges in their industries, and their resources gradually reallocate to those with lower carbon-control costs [55]. On the other hand, improvements in resource allocation efficiency also manifest as productivity gains [56,57]. LCCP incentivizes firms to optimize resource allocation and innovate technologies, bringing their input and output combinations closer to the optimal production frontier, which further boosts production efficiency [49]. Hence, LCCP not only raises firms’ production efficiency, which indicates better resource allocation. Through both constraining and incentivising mechanisms, it significantly enhances overall resource allocation efficiency. As resource allocation efficiency is a key driver of firm transformation and upgrading [58,59], LCCP thereby facilitates firms’ structural and technological upgrading.

Finally, LCCP can elevate firms’ positions in the value chain, thus further aiding their transformation and upgrading. A region’s technological level and production efficiency determine where its products are situated within the global value chain [48]. By fostering innovation and efficiency improvements, LCCP enables firms climb to higher value-chain segments. Moreover, environmental policies more broadly have been shown to promote upward movement in the value chain [60,61]. As a representative urban environmental policy in China, LCCP similarly enables firms to attain more advanced roles. When firms enter higher value-chain tiers, they undergo various forms of upgrading, which in turn accelerates their overall transformation [62].

Based on these considerations, this paper proposes the following hypotheses

Hypothesis 1: LCCP promote enterprise transformation and upgrading

### 2.2. Moderating role of enterprise green development level

LCCP most directly affects enterprise green development level [63]. Enterprise green development refers to a firm’s ability to achieve sustainable development. This involves balancing economic growth with environmental protection, improving the efficiency of environmental input–output, advancing green technological innovation, and strengthening environmental management [64–66]. Thus, enterprise green development level reflects the actual achievement levels across its various dimensions and serves as a key indicator for assessing the effectiveness of corporate sustainable development. The higher the enterprise green development level, the stronger the foundation for transformation and upgrading. Therefore, this study explores the mechanism how LCCP influences enterprise transformation and upgrading through enterprise green development level. According to its definition, the key elements of enterprise green development level mainly include GTFP, green innovation, and green management [64,67,68]. These three dimensions respectively capture the degree of enterprise green development in terms of efficiency, quality, and institutional guarantee.

In existing empirical studies, total factor productivity, innovation, and management are often used as mechanism variables to assess the impact of LCCP on firms [46,69–71]. However, few studies adopt a corporate green‑development perspective that harmoniously integrates GTFP, green innovation, and green management. They also rarely investigates the channels through which LCCP drive firm transformation and upgrading across the dimensions of efficiency, quality, and institutional safeguards. Therefore, this paper treats GTFP, green innovation, and green management as moderating variables, It uses this framework to explore how LCCP influence enterprise transformation and upgrading.

#### 2.2.1. Moderating role of enterprise green total factor productivity.

Green total factor productivity (GTFP) is a critical indicator of the quality of enterprise green development. GTFP incorporates environmental factors into the traditional total factor productivity framework to evaluate the efficiency of inputs and outputs in the nexus of economic growth and environmental impact. It therefore serves as an essential metric for assessing how to mitigate environmental pressures, enhance environmental performance, and secure sustainable economic development [72,73]. GTFP represents the green efficiency aspect of enterprise transformation and upgrading. A higher GTFP indicates that an enterprise achieves greater input–output efficiency while simultaneously maintaining robust environmental performance.

Higher GTFP can strengthen an enterprise’s adaptability to LCCP, thereby further enhancing its momentum and potential for transformation and upgrading. First, higher GTFP reduces the need for environmental protection investment, thus improving an enterprise’s policy adaptability. A higher GTFP indicates robust low-carbon governance capacity. This allows the firm to meet LCCP environmental standards with lower environmental expenditures and adjustment costs, and to more readily satisfy the eligibility criteria for associated preferential policies, securing financial and policy support. Consequently, when LCCP drives enterprise transformation and upgrading, higher GTFP helps firms better accommodate these changes.

Second, higher GTFP enhances green output efficiency, further boosting an enterprise’s policy adaptability. Elevated GTFP often reflects superior green output efficiency under constraints of resource consumption and pollutant emissions [74,75]. Thus, when LCCP imposes environmental constraints, firms with higher GTFP can maintain high output efficiency and leverage low-carbon incentives to foster better development. As a result, enterprises with high GTFP exhibit greater synergy with LCCP, facilitating more effective transformation and upgrading.

Based on the above, this study proposes the following hypothesis.

Hypothesis 2. Enterprise GTFP positively moderates the relationship between LCCP and enterprise transformation and upgrading.

#### 2.3.3. Moderating role of enterprise green innovation.

Enterprise green innovation is a key dimension of enterprise green development level, representing its qualitative aspect. Green innovation refers to hardware-based or software-based innovations related to green products or processes, including technologies for energy conservation, pollution control, waste recycling, green product design, or enterprise environmental management systems [76]. Green innovation enhances firms’ responsiveness to LCCP incentives and elevates the enterprise green development level, thereby strengthening their ability to achieve transformation and upgrading.

Green innovation improves transformation and upgrading capacity through two primary pathways. First, it builds the foundational capabilities for transformation and upgrading. By definition, green innovation integrates environmental friendliness with economic benefits in an innovation model that is vital for promoting sustainable development. Moreover, green innovation encompasses new technologies, goods, services, production processes, or management systems that reduce environmental pollution and contribute to sustainable development [77,78]. This comprehensive form of innovation equips firms with the essential capabilities to capitalize on opportunities from LCCP. It helps them achieve realize successful transformation and upgrading.

Second, the pursuit of green innovation enhances firms’ competitiveness and advances their position in the value chain, further facilitating transformation and upgrading. Enterprise green innovation has become a crucial tool for firms to expand market share and sustain long-term vitality: it helps improve market position, attract customers, deliver green services, and secure competitive advantages [79]. Such competitive advantages not only help firms navigate the uncertainties introduced by LCCP but also mitigate the upfront investment pressures associated with policy compliance. Therefore, green innovation amplifies the positive effect of LCCP on enterprise transformation and upgrading.

Based on the above, this study proposes the following hypothesis.

Hypothesis 3. Enterprise green innovation positively moderates the relationship between LCCP and enterprise transformation and upgrading.

#### 2.3.4. Moderating role of enterprise green management.

Enterprise green management is a pivotal dimension of enterprise green development level, representing the institutional foundation for sustainable practices. Green management constitutes an organization-wide philosophy and practice system. It emphasizes deep integration of environmental objectives with corporate strategy, leveraging innovation and continuous improvement to achieve sustainable development and competitive advantage [80,81]. By strengthening internal governance structures, green management elevates firms’ potential and motivation for transformation and upgrading under LCCP.

Implementing green management enhances enterprises’ intrinsic transformational capacity and attracts external support, thereby facilitating transformation and upgrading. First, from an institutional perspective, green management builds internal potential for upgrading. The natural resources‐based view suggests that firms capable of utilizing or conserving natural resources through management practices can realize performance gains from this capability [82]. Similarly, the organizational capabilities perspective argues that green management, as an internal capability, provides a sustainable competitive advantage [83]. Moreover, green management positively influences organizational structure, innovation capacity, human resources, and cost savings [84]. Thus, under LCCP, firms with strong green management are better equipped alleviate regulatory pressure and more readily satisfy policy support conditions, promoting transformation and upgrading.

Second, green management helps secure external legitimacy and resources. As an indispensable component of corporate strategy, green management not only drives financial performance but also aligns with environmental protection, enhancing market legitimacy and social recognition. This increased credibility can translate into higher product value and sales [85]. Consequently, firms with strong green management are better positioned to meet LCCP requirements, obtain additional policy support, and thereby amplify the positive effect of LCCP on enterprise transformation and upgrading.

Based on the above, this study proposes the following hypothesis.

Hypothesis 4. Enterprise green management positively moderates the relationship between LCCP and enterprise transformation and upgrading.

**Fig 1:**
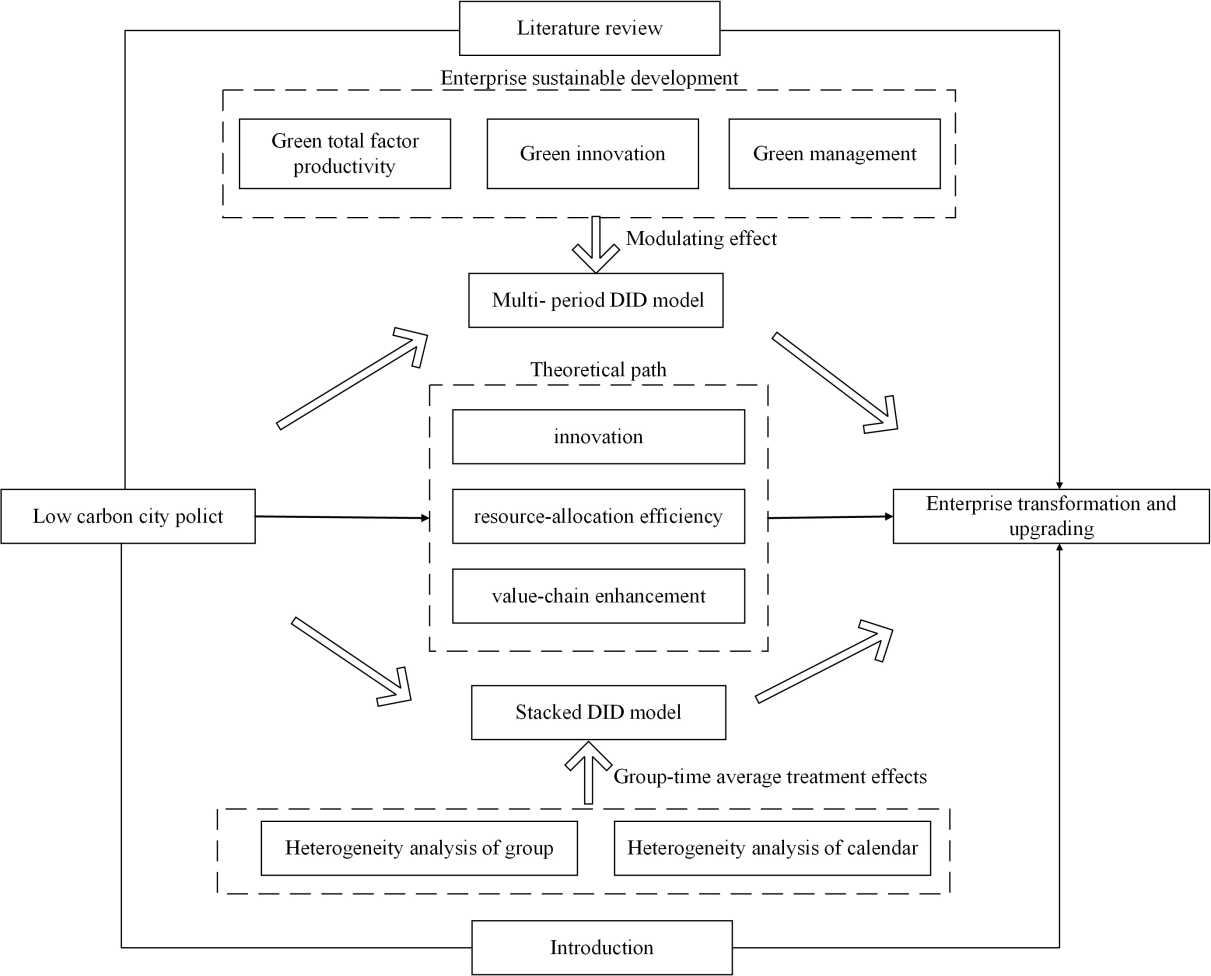
Theoretical framework.

Drawing on Lei et al. (2023) [86], as shown in Fig 1, this paper develops the theoretical framework of the present study. The theoretical framework of this paper is presented as follows. This study first employs a multi‑period DID model to examine the direct impact of LCCP on enterprise transformation and upgrading. Second, it augments the multi‑period DID specification by including interaction terms between the policy dummy and selected moderating variables. This helps investigate the channels through which LCCP influences firms’ transformation and upgrading. Finally, a stacked DID model is used to estimate group‑time average treatment effects.

## 3. Research methodology

### 3.1. Data

In 2009, following the United Nations Climate Change conference, actively responding to climate change became a major strategic priority for China’s socio-economic development. Accordingly, this study selects all A-share listed firms in China over the period 2009–2022 as its research sample, matching firm-level data with corresponding city-level indicators. All data are sourced from the China Stock Market & Accounting Research database (CSMAR) and the China National Research Data Service platform (CNRDS). From CSMAR we obtain total patent applications, invention patent applications, profits and taxes (PAT), labor productivity, total operating-cost ratio, cost-to-profit ratio, operating profit margin, return on capital, firm size, debt-to-asset ratio, return on net assets, firm age, proportion of independent directors, largest shareholder shareholding ratio, and management expense ratio; from CNRDS we collect green total-factor productivity, green innovation, green management, regional per-capita GDP, and regional industrialization level. Additionally, data on LCCP are hand collected.

For data preprocessing, we first clean the sample rigorously. We exclude firms with two consecutive years of losses or three consecutive years of losses, firms that have been delisted or suspended, firms with severely missing financial data, and firms in the financial or real-estate sectors. Second, to attenuate the impact of outliers on the empirical results, we winsorize all non-dummy variables at the 1st and 99th percentiles. Values below the 1st percentile are replaced with the 1st -percentile value, and values above the 99th percentile are replaced with the 99th-percentile value. The final dataset comprises 2,625 firms located in 278 cities.

### 3.2. Model specification

The core issue of this study is whether LCCP have promoted enterprise transformation and upgrading. Since the policy implementation in 2010, the National Development and Reform Commission has successively carried out the second and third rounds of LCCP work in 2012 and 2017. The multi-period DID model has been widely used in policy research in the field of environmental economics [87]. Considering that the policy was launched in three batches, this study follows the research by Beck et al. (2010) [88] and adopts a multi-period DID model for policy effect evaluation. The multi-period DID model considers policy implementation as an exogenous quasi-natural experiment, which effectively addresses endogeneity issues and removes the effects of time variations and other unmeasurable factors. This approach allows for a clear identification of the pilot policy’s impact on enterprise transformation and upgrading. The model is designed with listed companies in low-carbon pilot cities as the experimental group, and listed companies in non-low-carbon pilot cities as the control group.


$$\begin{array}{c} {Trans}_{i,t}=\alpha_{\text{0}}+\alpha_{\text{1}}{PLC}_{i,t}+\alpha_{\text{2}}\sum {Control}_{i,t}+\lambda_{i}+\delta_{j}+\mu_{i}+\varepsilon_{i,j,t} \end{array}$$ (1)

Here, *i, j* and *t* represent enterprises, cities, and time, respectively. ${Trans}_{i,t}$ is the dependent variable, which indicates enterprise transformation and upgrading; ${PLC}_{i,t}$ is the independent variable, a dummy variable distinguishing whether city j becomes a low-carbon city in year *t*; ${Control}_{i,t}$ denotes the control variables at the enterprise and city levels. The model also includes firm fixed effec*t*s, city fixed effects, and time fixed effects, with $\varepsilon_{i,j,t}$ being the unobservable random error term.

Equation (1) presents the multi-period DID specification. When firms participate in the policy at heterogeneous time points, the standard two period, two group DID design is no longer applicable; instead, we construct a multi-period difference-in-differences model. This framework allows us to assess the dynamic effects of a policy or event on treated firms across different implementation dates. Since the LCCP was rolled out in three successive waves, each announcing a new batch of participating cities at distinct time nodes. This naturally meets the requirement of variation in treatment timing. Accordingly, we employ a multi-period DID approach for our empirical analysis. By using this model, researchers can not only estimate the average treatment effect. They can also trace out pre-treatment trend tests and post-treatment dynamic effect curves. This approach thoroughly validating the parallel trends assumption, detecting any placebo effects before policy implementation, and quantifying the intensity and persistence of the policy’s impact at each stage.

Considering that different enterprises require distinct strategies for transformation and upgrading, clustering at the enterprise level helps identify and analyze the heterogeneity of the policy impact. Furthermore, enterprises may adopt a series of measures in response to LCCP, such as improving production processes or developing new technologies. These changes reflect the dynamic nature of internal decision-making and strategy adjustments, which may introduce time-series correlation in the error terms at the enterprise level. Therefore, this study employs a standard error calculation method clustered at the enterprise level.


$$\begin{array}{c} {Trans}_{i,t}=\alpha_{\text{0}}+\alpha_{\text{1}}{PLC}_{i,t}+\alpha_{\text{2}}{Moderation}_{i,t}+\alpha_{\text{3}}{PLC}_{i,t}\times {Moderation}_{i,t}+\alpha_{\text{3}}\sum {Control}_{i,t}+\lambda_{i}+\delta_{j}+\mu_{i}+\varepsilon_{i,j,t} \end{array}$$ (2)

${Moderation}_{i,t}$ denotes the moderating variable for firm *i* in year *t*. In the above model, we primarily assess whether the coefficien*t* $\alpha_{\text{3}}$ on the interaction term is statistically significant; a significant α₃ indicates the presence of a moderating effect.

### 3.3. Variable selection

#### 3.3.1. Enterprise transformation and upgrading.

To date, the measurement of enterprise transformation and upgrading can be classified into three categories. The first category relies on a single indicator. Total factor productivity is the most commonly used measure of upgrading effects, as it encompasses multiple dimensions such as technological progress and management capability [4,59]. Some studies, however, employ a firm’s position in the global value chain [89] or its technological sophistication to capture transformation and upgrading [13,90]. The second category adopts multiple indicators. For example, Wen et al. (2021) [3] and Xie et al. (2023) [91] separately measure enterprise transformation and upgrading through total factor productivity and innovation, reflecting the efficiency and quality dimensions. Other research uses value chain upgrading and green transformation as dual variables to assess transformation and upgrading [18]. The third category constructs composite indices. Pavlínek and Ženka (2011) [92] combine per-capita operating revenue, factor productivity, per-capita wages, and R&D investment into a single measure. More recently, Hu et al. (2023) [93] develop a comprehensive enterprise transformation and upgrading index by integrating indicators of innovation capacity, operational efficiency, and value chain advancement.

Despite the popularity of single indicator measures, owed to their operational simplicity and intuitive interpretation, they have clear drawbacks: they capture only one facet of upgrading and cannot simultaneously reflect technological progress, management efficiency, and industrial status. Although parallel use of multiple indicators offers a fuller depiction, it faces two challenges: (1) absence of a standardized weighting and aggregation framework makes objective determination of indicator importance difficult; and (2) lack of synergy among indicators undermines the derivation of a cohesive overall evaluation. To overcome these limitations, this study follows Hu et al. (2023) [93] by focusing on three dimensions, innovation capacity, product value addition, and operational efficiency, which are highly relevant to Chinese firms. It constructs a composite index via the entropy weighting method.

As shown in Table 1, innovation capacity is proxied by total patent applications and invention patent applications; operational efficiency is captured through cost effectiveness (labor productivity, operating cost, and profitability) and profitability (operating profit margin and return on total assets); and product value addition is represented by tax paid earnings (post tax profit plus taxes and surcharges). The entropy weighting procedure is as follows. First, classify the indicators into positive indicators (where higher values are better) and negative indicators (where lower values are better), and then perform dimensionless normalization. Next, express each firm’s normalized value for a given indicator as a proportion of the sum of that indicator across all firms. Then compute the information entropy of each indicator, derive the dispersion coefficient from the entropy, and determine the entropy weight based on that coefficient. Finally, apply these weights to the normalized values to obtain each firm’s composite score across all indicators, yielding a comprehensive index of enterprise transformation and upgrading.

**Table 1:** Variables and their interpretations.

	Variable	Indicator		Specific Definition
Dependent Variable	Comprehensive Index of Enterprise Transformation and Upgrading	Innovation Capability	Total Patent Applications	Total number of patents independently applied by the company in the year
			Total Invention Patent Applications	Total number of invention patents independently applied by the company in the year
		Product Added Value	Taxes and Profits	Main business tax and additional charges + total profit
		Taxes and Profits	Labor Productivity	Ln(total profit/number of employees at year-end)
			Total Operating Cost Rate	Total operating cost/total operating income
			Cost-Expense Profit Rate	Total profit/total operating cost
			Operating Profit Rate	Operating profit/total operating income
			Return on Capital	Total profit/(fixed assets + current assets)
Independent Variable	LCCP	Dummy variable for whether LCCP is implemented		If the company’s location city is a low-carbon pilot city in the current year or thereafter, it takes a value of 1; otherwise, it takes a value of 0.
Moderator Variable	Enterprise Green Development Quality	GTFP		Measured using the Malmquist-Luenberger Index
		Green Innovation		Ln(number of green invention patent applications) Ln(number of green invention patent applications)
		Green Management		Comprehensive score involving green management
Control Variables	At the Enterprise Level	Enterprise Scale		Natural logarithm of total assets
		Asset-Liability Ratio		Total liabilities/total assets
		Return on Equity		Net profit/average net assets
		Company Age		Observation year minus year of establishment plus 1
		Proportion of Independent Directors		Number of independent directors/number of directors
		Shareholding Ratio of the Largest Shareholder		Shares held by the largest shareholder/total shares
		Management Expense Ratio		Management expenses/main business income
	At the Regional Level	Regional Per Capita GDP		Natural logarithm of regional per capita GDP
		Regional Industrialization Level		Industrial added value/regional GDP

The entropy weighting method objectively captures information disparities among sub indicators while avoiding subjective bias in weight assignment, making it well suited for multidimensional, heterogeneous indicator systems.

#### 3.3.2. LCCP.

The core explanatory variable is the dummy variable for low-carbon cities (PLC). Referring to the research of Cheng et al. (2019) [31], it is determined whether the city *j* where the enterprise is located implements the LCCP in year *i*. If the city is a low-carbon pilot city in the current year and thereafter, the value is 1; otherwise, it is 0. When dividing the experimental and control groups, there is an overlap between the pilot areas at the provincial and prefecture levels. If the province’s initiation time is earlier than the separate initiation time of the prefecture-level city, the provincial initiation time is taken as the standard. The data related to LCCP were manually collected using the following methods. Since LCCP is a policy issued by the National Development and Reform Commission, we gathered information on the pilot cities from 2010, 2012, and 2017 from the official website of the National Development and Reform Commission, and used this information to construct the explanatory variables.

#### 3.3.3. Control variables.

This paper controls for variables that may affect enterprise transformation and upgrading at both the enterprise and regional levels. At the enterprise level, following the study by Luo et al. (2022) [94], the following indicators are selected to measure the operational status of enterprises: enterprise size (Size), asset-liability ratio (Lev), return on equity (ROE), and firm age (FirmAge). To measure managerial ability, the following indicators are chosen: proportion of independent directors (Indep), shareholding ratio of the largest shareholder (Top1), and management expense ratio (MFR). At the regional level, referring to Yin et al. (2023) [95], the following indicators are controlled for: regional per capita GDP (RGDP) and regional industrialization level (Industry). Given that some of the control variables have a significant amount of missing data, interpolation methods are employed to complete the ROE data, ensuring data continuity.

#### 3.3.4. The quality of enterprise green development.

The moderating variables are the quality of enterprise green development, divided into three indicators: GTFP (GTFP), green innovation, and green management.

GTFP (GTFP) is measured based on the Malmquist-Luenberger index, following Wu et al. (2022) [96]. Factor inputs include labor input, represented by the number of employees; capital input, represented by the net value of fixed assets; and energy input, represented by industrial electricity consumption converted based on the proportion of employees in the city. The expected output is represented by operating income, and the non-expected output is represented by “industrial three wastes” (industrial sulfur dioxide, industrial wastewater, and industrial dust emissions), converted based on the proportion of employees in the city. The ML index reflects the change rate of GTFP, with 2008 as the base year (set to 1), and combined with the ML index calculations, the GTFP indicators for A-share listed companies from 2009 to 2022 are obtained.

Green innovation is represented by the natural logarithm of the number of green invention patent applications and the natural logarithm of the total number of green invention and utility model patent applications.

Green management, following the research of Xi and Zhao (2022) [97], is measured by whether the company has ISO14001 and ISO9001 certifications, and by adding up scores from the environmental management disclosure in the company’s annual report, including environmental management systems, environmental education and training, and special environmental actions.

As shown in Table 1, this study provides a summary of the main variables employed and their corresponding definitions.

## 4. Empirical analysis

### 4.1. Descriptive statistics

As shown in Table 2, this study summarizes the core characteristics of the sample data through descriptive statistics. The mean of enterprise transformation and upgrading (Trans) is 0.088, indicating that Chinese firms exhibit a low degree of transformation and upgrading with the process being relatively slow. The standard deviation of Trans is 0.141, with values ranging from 0.015 to 0.984, reflecting substantial heterogeneity in the extent of enterprise transformation and upgrading across firms. This suggests that firm‑internal incentives and market‑driven forces alone are insufficient to drive large‑scale, deep‑level transformation and upgrading, emphasizing the need for external policy stimuli.

**Table 2:** Descriptive statistics.

Variable	Obs.	Mean	Median	S.D.	Minimum	Maximum
Trans	20342	0.088	0.044	0.141	0.015	0.984
treat	20342	0.711	1.000	0.453	0.000	1.000
post	20342	0.584	1.000	0.493	0.000	1.000
PLC	20342	0.584	1.000	0.493	0.000	1.000
Size	20342	22.46	22.27	1.297	19.32	26.45
Lev	20342	0.437	0.438	0.189	0.027	0.908
ROE	20342	0.070	0.078	0.108	−0.827	0.644
FirmAge	20342	2.865	2.944	0.372	1.099	3.611
Indep	20342	37.30	33.33	5.304	25.00	60.00
Top1	20342	36.03	34.04	15.08	8.020	75.84
MFR	20342	0.079	0.064	0.069	0.006	0.937
RGDP	20342	11.40	11.47	0.557	8.704	13.06
Industry	20342	0.348	0.370	0.088	0.070	0.574

The mean of the LCCP variable (LCCP) is 0.584, denoting that more than half of the sampled firms participate in LCCP, which implies that LCCP has become relatively widespread and has a significant impact. The means of firm size (Size), leverage ratio (Lev), and return on equity (ROE) are 22.46, 0.437, and 0.070, respectively, with corresponding standard deviations of 1.297, 0.189, and 0.108, indicating that most firms maintain stable operations and possess a foundational capacity for transformation and upgrading. Finally, the means of regional GDP (RGDP) and regional industrialization level (Industry) are 11.40 and 0.348, respectively, with standard deviations of 0.557 and 0.088, suggesting notable inter‑regional disparities in economic development.

### 4.2. Hypothesis test results

#### 4.2.1. LCCP and enterprise transformation and upgrading.

To test Hypothesis H1, we first perform a preliminary estimation of model (1) using Pooled OLS to examine the relationship between LCCP and enterprise transformation and upgrading. As shown in column (1) of Table 3, both PCL and Trans have a significantly positive correlation when using Pooled OLS with control variables included.

**Table 3:** The impact of LCCP on enterprise transformation and upgrading.

	Pooled OLS	Multiple high dimensional fixed effects regression		
	(1)	(2)	(3)	(4)
Variable	Trans	Trans	Trans	Trans
PLC	0.011***	0.011**	0.012***	
	(6.361)	(2.261)	(2.710)	
L.PLC				0.010**
				(2.286)
Size	0.053***		0.041***	0.042***
	(38.301)		(7.365)	(6.738)
Lev	−0.030***		−0.063***	−0.063***
	(−6.241)		(−4.631)	(−4.162)
ROE	0.059***		−0.003	−0.005
	(7.124)		(−0.277)	(−0.453)
FirmAge	−0.026***		0.040*	0.046
	(−9.879)		(1.706)	(1.645)
Indep	0.002***		−0.000	−0.000
	(7.938)		(−0.629)	(−0.526)
Top1	−0.000***		0.000	0.000
	(−5.151)		(0.203)	(0.049)
MFP	0.055***		−0.058***	−0.054**
	(5.098)		(−2.604)	(−2.059)
RGDP	0.012***		−0.008	−0.005
	(8.059)		(−1.343)	(−0.906)
Industry	−0.067***		0.072	0.073
	(−5.474)		(1.077)	(1.033)
Constant	−1.202***	0.083***	−0.855***	−0.928***
	(−35.289)	(30.306)	(−5.310)	(−5.040)
Firm FE	YES	YES	YES	YES
Year FE	YES	YES	YES	YES
City FE	YES	YES	YES	YES
Obs.	20,342	20,342	20,342	16350
Adj.R^2^	0.242	0.744	0.756	0.782

Note: *, **, and *** indicate statistical significance at the 10%, 5%, and 1% levels, respectively.

The tstatistics are given in parentheses, and the coefficients are based on standard errors clustered at the firm level.

Next, we use high-dimensional fixed effects estimation. As shown columns (3) and (4) of Table 3, PCL and Trans maintain a significant positive correlation both without control variables and with them. The coefficients are 0.011 and 0.012, respectively, and the influence of PCL on Trans increases after adding control variables. In summary, with the implementation of the LCCP has led enterprises to actively drive transformation and upgrading. Therefore, this evidence supports Hypothesis 1.

We also used a one-year lagged PLC (L.PLC) to preliminarily examine the dynamic effect of the LCCP. As shown in column (4) of Table 3, L.PLC and Trans still have a significantly positive correlation, although the coefficient decreases by 0.002. This indicates that the influence of the LCCP on enterprise transformation and upgrading diminishes in the following year. However, it still demonstrates that the LCCP exerts a dynamic effect on enterprise transformation and upgrading.

### 4.3. Endogeneity test

#### 4.3.1. Parallel trend test.

A fundamental prerequisite for using the DID model is that the parallel trend assumption must be satisfied. This means that the treatment group should maintain a similar parallel trend as the control group at the same time point before being designated as low-carbon cities. This ensures the fairness and validity of the estimated results. To verify this, this paper conducts a parallel trend test, referencing the study by Bunderson and Sutcliffe (2002). It is important to note that in the parallel trend test for multiple time points DID, different periods can represent different treatment periods rather than specific years. The period “before n” (n = 1, 2,...) represents the nth year before the policy intervention.

Enterprise transformation and upgrading is a complex and long-term process. The LCCP, as an environmental regulation, may initially increase costs for enterprises, but by promoting enterprise innovation and improving efficiency, these cost increases can be partially offset, thereby aiding in enterprise transformation and upgrading. Therefore, we believe that the positive impact of the LCCP on enterprise transformation and upgrading has a certain lag. Consequently, this study chooses eleven periods post-policy intervention for the treatment group to conduct the parallel trend test. Data from three periods before and eleven periods after the intervention are selected.

**Fig 2:**
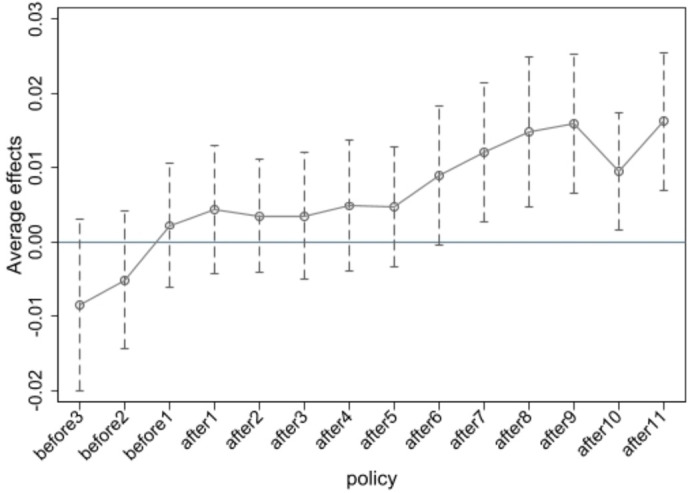
Parallel test.

As shown in Fig 2, the evolution of the degree of transformation and upgrading for both the treatment group and the control group at the same time point is consistent. This indicates that the parallel trend test is passed. The regression coefficients of the interaction terms are significantly positive six years after the establishment of the pilot. This demonstrates that the LCCP has a delayed positive effect on promoting enterprise transformation and upgrading.

#### 4.3.2. Placebo tests.

One possible scenario is that the promotion of enterprise transformation and upgrading is driven by unobservable random factors rather than the LCCP. In such a case, the baseline regression results would be a “spurious regression.”. Referencing Chetty et al. (2009) [98], to rule out these possibilities and ensure the reliability of the findings, we conduct placebo tests.

**Fig 3:**
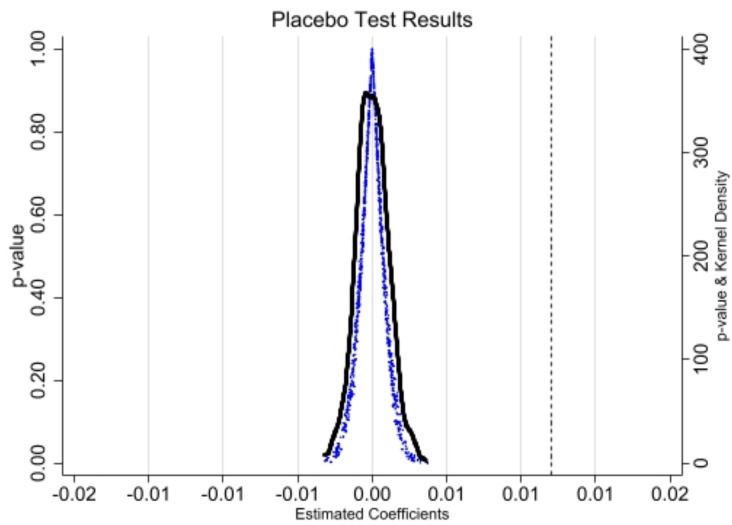
Placebo test.

Fig 3 shows that the coefficient from the baseline regression is 0.193, which is entirely independent of the coefficients from the random simulations. The coefficients from the random simulations are approximately 0. This demonstrates that the study results are not driven by random variables.

#### 4.3.3 PSM-DID.

**Fig 4:**
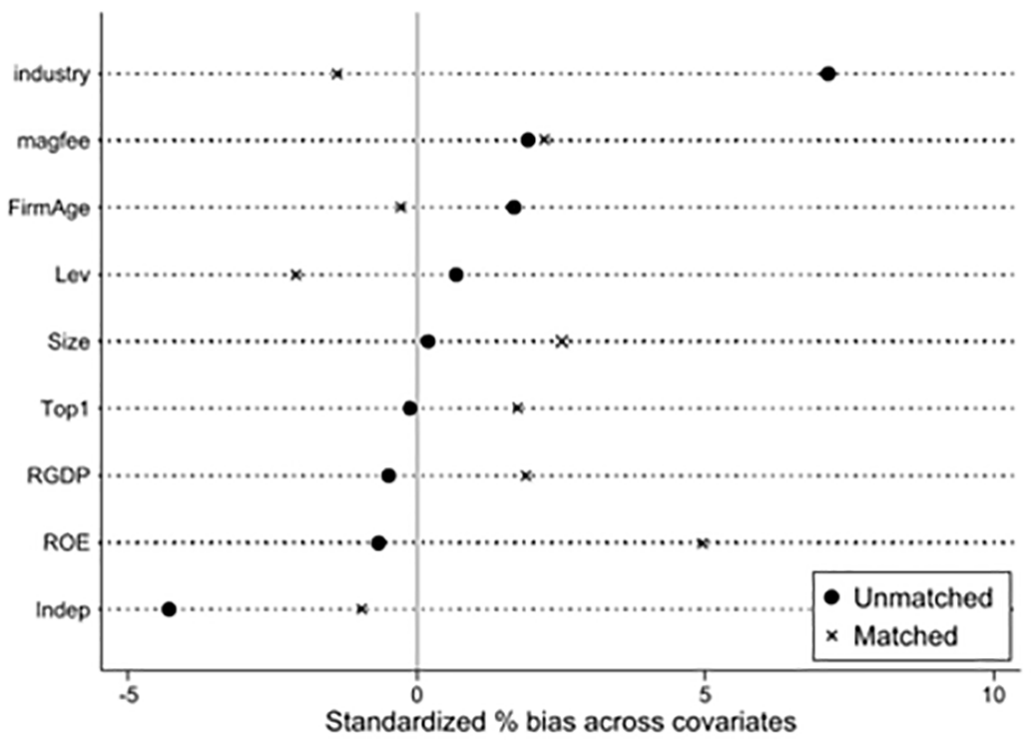
Propensity score matching.

**Fig 5:**
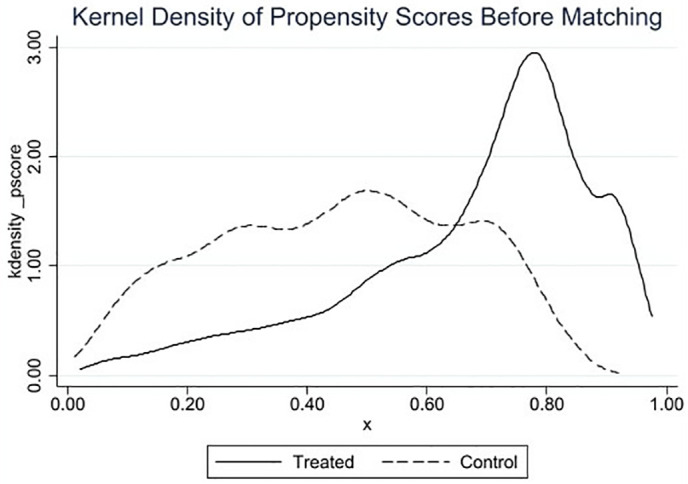
The kernel density curve (before).

**Fig 6:**
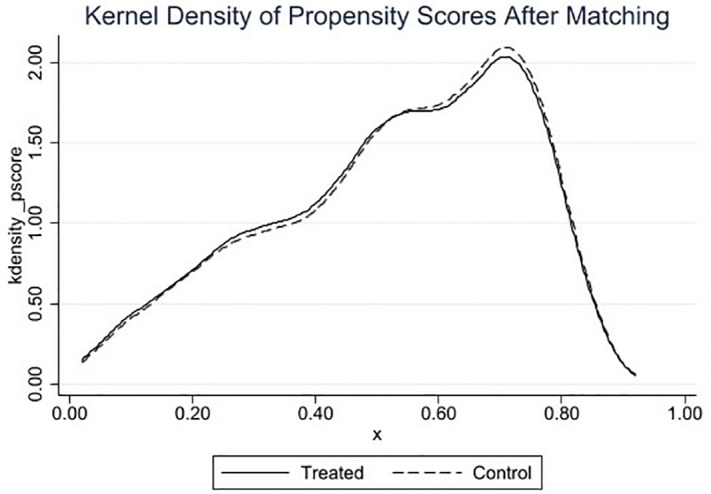
The kernel density curve (after).

This study employs the Propensity Score Matching-Difference-in-Differences (PSM-DID) model to test for selection bias in the control group sample in the baseline regression, which could lead to instability in the results. The propensity score matching method (1:1 nearest neighbor matching) is used to further filter the control group sample, reducing group variance between the experimental and control groups. As shown in Fig 4, a comparison of the standardized bias of the variables before and after matching shows a significant reduction in the standardized bias of the covariates post-matching. It also examines the common support region. As shown in Fig 5 and Fig 6, the kernel density function distribution plots indicate that the kernel density curves of the experimental group and the control group are closer after matching. The fitting effect is better than before matching, allowing for regression analysis. This indicates that after matching the covariates, they pass the balance test, and the matching effect is good, suggesting that the sample grouping design is more reasonable. As shown in Table 4, the results of the covariate balance tests for all variables before and after matching are listed. After the above processing, the test results for differences between groups indicate that there are no substantial differences in control variables between the experimental and control group samples. The kernel density curve of the propensity score value probability for the control group after matching is closer to that of the experimental group than before matching. This indicates an improved matching effect. Therefore, it is reasonable to use the PSM-DID method for robustness testing. As shown in column (1) of Table 4, after performing propensity score matching and then estimating Model 1, the results still show a significant positive correlation.

**Table 4:** Endogeneity test.

	(1)	(2)
	PSM-DID	IV
Variables	Trans	Trans
PLC	0.010**	0.178***
	(2.005)	(3.980)
Size	0.041***	0.044***
	(4.528)	(7.509)
Lev	−0.051**	−0.068***
	(−2.405)	(−4.499)
ROE	0.003	−0.005
	(0.239)	(−0.437)
FirmAge	0.018	0.034
	(0.780)	(1.485)
Indep	−0.000	−0.000
	(−0.090)	(−0.513)
Top1	0.000	−0.000
	(1.067)	(−0.207)
MFP	−0.041	−0.073***
	(−1.127)	(−2.638)
RGDP	−0.011	−0.019***
	(−1.277)	(−2.767)
industry	−0.119*	0.138*
	(−1.846)	(1.676)
Constant	−0.699***	
	(−3.196)	
Firm FE	YES	YES
Year FE	YES	YES
City FE	YES	YES
Kleibergen-Paap rk LM statistic		108.2***
Kleibergen-Paap rk Wald F statistic		109.26
Obs.	7887	19460

Note: *, **, and *** indicate statistical significance at the 10%, 5%, and 1% levels, respectively.

The tstatistics are given in parentheses, and the coefficients are based on standard errors clustered at the firm level.

#### 4.3.4. Instrumental variable method.

To address potential endogeneity arising from omitted variables, this study adopts the green coverage area in built-up regions as an instrumental variable, following the approach of Jing (2021) [99]. The urban area green coverage ratio is defined as the proportion of green space within those parts of a city that have been fully developed and are served by municipal utilities and infrastructure; it is computed by dividing the green space area by the total area of the developed urban zone. Urban greening can sequester carbon effectively and thereby reduce carbon dioxide emissions [100]. Because this ratio captures a city’s capacity for greening and its commitment to environmental protection, it serves as a useful indicator in the identification of cities with low carbon emissions. However, urban greening per se is unlikely to exert a direct influence on enterprise transformation and upgrading. According to the resource‑based view, a firm’s competitive advantage and ability to upgrade depend on resources and capabilities that are unique, valuable, and difficult to imitate. Enhanced urban greening can improve livability and public welfare. However, it is not a strategic resource that firms can directly deploy. It also does not inherently strengthen their technological expertise, managerial capacity, or organizational learning. For these reasons, the green coverage area in built-up regions is chosen as the instrumental variable in this analysis.

As shown in column (1) of Table 4, the Kleibergen-Paap rk LM test results are significant at the 1% level, indicating that the instrumental variable passes the underidentification test. The Kleibergen-Paap rk Wald F statistic is 109.26, much higher than the critical value of 16.38, indicating that the instrumental variable passes the weak identification test. After performing the instrumental variable regression, the effect of PLC on Trans remains significantly positive at the 1% level, thereby confirming the robustness of the study’s results.

### 4.4. Robustness tests

#### 4.4.1. Replacing the explained variable.

In this study, the Entropy Weight TOPSIS method is used to reconstruct the enterprise transformation and upgrading index (Trans_t). Specifically, the entropy weight method is used to determine the weights of each evaluation indicator to build a weighted decision matrix. Each element of the original decision matrix is multiplied by the corresponding indicator weight. The TOPSIS method steps are then applied to the weighted decision matrix to calculate the relative closeness and ranking of the schemes. The indicator calculated by the Entropy Weight TOPSIS method is used as the explained variable to re-estimate Model 1. As shown in column (1) of Table 5, the effect of PLC on Trans_t remains significantly positive at the 5% level.

**Table 5:** Robustness test.

	(1)	(2)	(3)
Variables	Trans_t	Trans	Trans
PLC	0.002**	0.012***	0.012**
	(2.426)	(2.643)	(2.005)
Size	0.001	0.042***	0.041***
	(1.308)	(7.304)	(6.774)
Lev	−0.009***	−0.065***	−0.063***
	(−3.201)	(−4.627)	(−2.716)
ROE	−0.007***	−0.005	−0.003
	(−4.524)	(−0.550)	(−0.274)
FirmAge	0.025**	0.044*	0.040
	(2.484)	(1.786)	(0.787)
Indep	−0.000	−0.000	−0.000
	(−0.774)	(−0.603)	(−0.763)
Top1	−0.000	0.000	0.000
	(−0.737)	(0.110)	(0.174)
MFR	−0.008**	−0.060***	−0.058**
	(−2.263)	(−2.666)	(−2.434)
RGDP	0.002**	−0.008	−0.008
	(1.985)	(−1.254)	(−1.624)
industry	0.037**	0.077	0.072
	(2.035)	(1.097)	(0.590)
did1		−0.012	
		(−1.598)	
did2		−0.001	
		(−0.268)	
Constant	−0.100***	−0.894***	−0.855***
	(−3.042)	(−5.286)	(−3.108)
Firm FE	YES	YES	YES
Year FE	YES	YES	YES
City FE	YES	YES	YES
cluster	enterprises	enterprises	city
Obs.	20,342	19,033	20,342
Adj.R^2^	0.553	0.758	0.756

Note: *, **, and *** indicate statistical significance at the 10%, 5%, and 1% levels, respectively. The tstatistics are given in parentheses, and the coefficients are based on standard errors clustered at the firm lev-el(except (3)).

#### 4.4.2. Adding other policies as control variables.

During the implementation of LCCP, China also implemented other environmental policies such as the green credit policy and the carbon emissions trading policy. These could affect enterprise environmental decisions or environmental performance, potentially affecting the results of this study. First, after the introduction of the “Green Credit Guidelines” in 2012, green credit has played an important role in promoting green development. The green credit policy encourages companies to reduce energy consumption and save resources while integrating environmental factors into the financial accounting and credit decision-making processes of the financial industry. A company’s low-carbon behavior affects its capital costs, thereby restricting or supporting its transformation and upgrading at the funding level. To account for this, we construct a dummy variable for the green credit policy by treating all firms established after 2012 as having participated in green credit [101]. We include the interaction term did1 as a control. It is the product of the green credit policy-year dummy and the LCCP pilot-region firm dummy. (i.e., the product of the dummy for whether a firm participates in the green credit policy and the dummy for whether it participates in the LCCP).

Next, the carbon emissions trading policy is a market‑based environmental regulation tool. It aims to steer firms toward lower carbon emissions via market mechanisms and influences their environmental strategy decisions. Since October 2011, carbon emissions trading pilots have been launched, with Beijing, Shanghai, Tianjin, Chongqing, Hubei, Guangdong, and Shenzhen approved as pilot regions for carbon emissions trading preparatory work. We define the dummy for participation in the carbon emissions trading policy as equal to 1 if, after 2012, a firm is located in one of those seven regions, and 0 otherwise [102]. We include the interaction term did2 as a control. It is the product of the carbon emissions trading policy-year dummy and the LCCP pilot-region firm dummy (i.e., the product of the dummy for whether a firm participates in the carbon emissions trading policy and the dummy for whether it participates in the LCCP).

As shown in column (2) of Table 5, after introducing the interaction terms of the two policies as control variables, the effect of PLC on Trans remains significantly positive at the 1% level. This indicates that the impact of LCCP on enterprise transformation and upgrading is robust.

#### 4.4.3. Other robustness tests.

This study uses city-clustered standard errors to re-estimate Model 1. As shown in column (3) of Table 5, when using city-clustered standard errors, the results remain significantly positive at the 5% level. This demonstrates the robustness of the impact of LCCP on enterprise transformation and upgrading.

## 5. Heterogeneity analysis

### 5.1. Heterogeneity analysis of enterprise green development level

The aforementioned studies indicate that LCCP help promote enterprise transformation and upgrading. In line with hypotheses H2, H3, H4, and H5 proposed in this paper, the following sections will focus on examining whether the moderating effect of enterprise green development level exists.

As shown in Table 6, columns (1), (2), (3), (4), and (5) present the estimation results for Model 2, Model 3, Model 4, and Model 5, respectively. The interaction term between LCCP (PLC) and GTFP (GTFP) in column (1) has an estimated coefficient of 0.01, which is significantly positive at the 1% level. This suggests that higher enterprise GTFP enhances the promoting effect of LCCP on enterprise transformation and upgrading.

**Table 6:** Heterogeneity analysis of enterprise green development quality.

	(1)	(2)	(3)	(4)
Variables	Trans	Trans	Trans	Trans
PLC	0.015***	0.007*	0.009**	0.013***
	(3.048)	(1.928)	(2.549)	(2.869)
GTFP	−0.009*			
	(−1.808)			
GTFP* PLC	0.010***			
	(3.422)			
Ginv1		0.046***		
		(9.576)		
Ginv1* PLC		0.026***		
		(5.328)		
Ginv2			0.037***	
			(8.495)	
Ginv2* PLC			0.032***	
			(6.739)	
Gmag				−0.006***
				(−3.634)
Gmag* PLC				0.015***
				(6.733)
Size	0.042***	0.022***	0.022***	0.043***
	(7.156)	(5.266)	(4.985)	(7.167)
Lev	−0.058***	−0.040***	−0.044***	−0.069***
	(−4.118)	(−3.779)	(−3.920)	(−4.650)
ROE	−0.001	0.008	0.003	−0.006
	(−0.118)	(0.989)	(0.421)	(−0.507)
FirmAge	0.040	0.028	0.030*	0.041
	(1.517)	(1.622)	(1.659)	(1.631)
Indep	−0.000	−0.000	−0.000	−0.000
	(−0.457)	(−1.566)	(−1.423)	(−0.657)
Top1	−0.000	0.000	0.000	0.000
	(−0.035)	(0.278)	(0.511)	(0.187)
MFR	−0.052**	−0.023	−0.031*	−0.075***
	(−2.331)	(−1.460)	(−1.853)	(−3.193)
RGDP	−0.006	−0.000	−0.000	−0.006
	(−0.920)	(−0.012)	(−0.075)	(−0.982)
industry	0.058	0.026	0.038	0.069
	(0.855)	(0.510)	(0.693)	(0.947)
Constant	−0.893***	−0.470***	−0.487***	−0.905***
	(−5.185)	(−3.764)	(−3.669)	(−5.441)
Firm FE	YES	YES	YES	YES
Year FE	YES	YES	YES	YES
City FE	YES	YES	YES	YES
Obs.	18,910	20,342	20,342	17,636
Adj.R^2^	0.761	0.822	0.809	0.765

Note: *, **, and *** indicate statistical significance at the 10%, 5%, and 1% levels, respectively.

The tstatistics are given in parentheses, and the coefficients are based on standard errors clustered at the firm level.

Table 6 columns (2) and (3) examine the moderating role of green innovation. The interaction terms between enterprise green invention patent applications (Ginv1) and overall green patent applications (Ginv2) with LCCP (PLC) are significantly positive at the 1% level, indicating that green innovation positively moderates the effect of LCCP on enterprise transformation and upgrading.

Table 6 column (4) presents the mechanism test results for enterprise green management, showing that the interaction term between green management (Gmag) and LCCP (PLC) is significantly positive. The estimated coefficient passes the 1% significance test, indicating that green management also has a positive moderating effect.

In summary, the positive moderating effect of enterprise green innovation is the strongest, followed by GTFP. The regression results in Table 6 validate the mechanism through which LCCP influence enterprise transformation and upgrading. Enterprise green development quality enhances the promoting effect of LCCP on enterprise transformation and upgrading.

The statistics are given in parentheses, and the coefficients are based on standard errors clustered at the firm level.

### 5.2. Heterogeneity analysis of group and time

The LCCP was implemented at three different points in time (2010, 2012, and 2017). At each of these points, new enterprises joined the pilot policies, indicating that different enterprises were affected by the policies at different times. This means the experimental group is dynamically changing over time, fitting the characteristics of staggered DID. Following the research of Callaway and Sant’Anna (2021) [103], this study employs a DID model with multiple time periods to estimate the Average Treatment Effects on the Treated (ATT) of LCCP on enterprise transformation and upgrading. This allows for the examination of both the heterogeneous effects across different groups (Heterogeneity analysis of group) and the dynamic effects over time (Heterogeneity analysis of time).

Firstly, enterprises were divided into three groups based on the three time points of the LCCP: those participating in 2010, 2012, and 2017. This analysis explores whether the timing of participation in LCCP influences the degree of enterprise transformation and upgrading differently. Secondly, the study estimates the effects by policy year to analyze whether the LCCP have different impacts on enterprise transformation and upgrading at different times.

**Fig 7:**
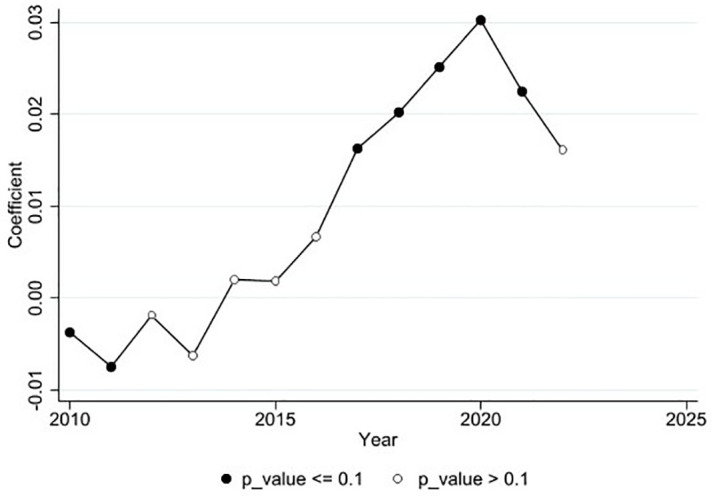
Dynamic effect.

As shown in Table 7, column (1) presents the dynamic effects of LCCP on enterprise transformation and upgrading over a 13-year period from the first pilot in 2010. Column (2) presents the multi-period treatment effects based on the policy time points, dividing the data into three groups. As shown in Fig 7, the line graph shows the dynamic trend from 2010 to 2022, with solid dots indicating years where Trans and PLC are significantly related and hollow dots indicating years where they are not.

**Table 7:** The Average Treatment Effects on the Treated.

(1)		(2)	
Year	Trans	Group	Trans
T2010	−0.004*	G2010	−0.002
T2011	−0.008**	G2012	0.025***
T2012	−0.002	G2017	0.015*
T2013	−0.006		
T2014	0.002		
T2015	0.002		
T2016	0.007		
T2017	0.016*		
T2018	0.020**		
T2019	0.025***		
T2020	0.030***		
T2021	0.023**		
T2022	0.016		
Obs.	15156	Obs.	15156
Pretrend Test H0 All Pre-treatment are equal to 0		p-value = 0.518	

Note: *, **, and *** indicate statistical significance at the 10%, 5%, and 1% levels, respectively.

From Table 7, column (1), and Fig 7, Since the first implementation of the LCCP in 2010, the policy’s impact on enterprise transformation and upgrading shows an initial significant negative correlation. It then becomes non-significant and eventually shows a significant positive correlation. This aligns with the Porter Hypothesis, suggesting that environmental regulation policies initially increase enterprise costs but also compel enterprises to innovate and improve efficiency to meet regulatory requirements. This process helps reduce resource dependence and pollution. It also potentially lowering production costs or creating new market opportunities, thus promoting enterprise transformation and upgrading.

From Table 7, column (2), it is evident that the policy’s impact on enterprise transformation and upgrading is not significant for those enterprises that participated in the LCCP in 2010. This may be due to the lack of experience during the initial phase, leading to imperfect pilot policies and unreasonable environmental regulations. Consequently, cities had to innovate LCCP to build low-carbon cities, and the risks and costs of policy innovation, being externalities, spread to the enterprises, affecting their transformation and upgrading.

However, for enterprises in the 2012 and 2017 groups, the coefficients are 0.025 and 0.015, respectively, and are significantly positive at the 1% and 10% levels. This indicates that participating in the LCCP in 2012 and 2017 promotes enterprise transformation and upgrading. This positive effect may be attributed to the lessons learned from previous pilot phases, which led to more reasonable environmental regulations. The 2017 policy implementation shows a decrease in both the coefficient and significance compared to 2012. This might be due to the introduction of many environmental protection and LCCP after China joined the Paris Agreement at the end of 2016 (e.g. green credit policies and carbon emission trading policies). These environmental policies have certain impacts on enterprises, thereby somewhat diminishing the promotional effect of the LCCP on enterprise transformation and upgrading. Additionally, since enterprise transformation and upgrading is a long-term process, enterprises that joined the LCCP in 2017 may not have fully progressed in their transformation by that time.

The null hypothesis (H0) of this test is that all pre-treatment effects are zero, meaning there should be no significant differences in the transformation and upgrading trends between the treatment and control groups before the LCCP was implemented. As shown in Table 7, the p-value is 0.518, indicating the hypothesis is not established, which aligns with the parallel trends assumption.

## 6. Conclusion

This paper focuses on the impact of LCCP on enterprise transformation and upgrading. Using a multi-time-point DID method, empirical analysis is conducted on A-share listed companies in China. This study explores the promoting effect of the implementation of LCCP on enterprise transformation and upgrading, along with the underlying mechanisms.

Firstly, this study finds that the LCCP has a positive promoting effect on enterprise transformation and upgrading. The comprehensive index of enterprise transformation and upgrading increasing by 0.012 for companies participating in the LCCP. Further analysis, including parallel trend testing, reveals that this effect exhibits a certain degree of lag after the policy’s implementation. However, in the long term, the implementation of the LCCP continues to have a sustained positive impact on enterprise transformation and upgrading.

Second, this paper integrates the enterprise green development level into the analytical framework of LCCP’s impact on enterprise transformation and upgrading. We comprehensively examine the moderating roles of GTFP, green innovation, and green management. The findings indicate that the enterprise green development level positively moderates the promoting effect of the pilot policy on enterprise transformation and upgrading.

Lastly, both theoretical analysis and empirical evidence reveal the dynamic effects and heterogeneous promoting effects of low-carbon transformation pilot policies on enterprise transformation and upgrading at different time points. Specifically, the promoting effect of the LCCP on enterprise transformation and upgrading strengthens over time, with the effects being more pronounced for enterprises that participated in the policy in 2012 and 2017.

In practical terms, the impact of the LCCP on enterprise transformation and upgrading holds significant reference value. Firstly, since the implementation of the LCCP, 95% of pilot cities have successfully reduced their carbon intensity significantly. By 2019, two years after the completion of the third batch of low-carbon city construction, China’s carbon intensity had decreased by 48.1% compared to 2005 levels, achieving the 2020 greenhouse gas emission control target [104,105]. This indicates that the LCCP effectively controlled carbon emissions. More importantly, the LCCP not only constrained carbon emissions but also significantly promoted enterprise transformation and upgrading. It achieved this by altering the institutional environment in which enterprises operate. This promoting effect exhibits a certain degree of lag and long-term sustainability. Therefore, policy-making should adopt a phased approach and tailor strategies to different cities. It should dynamically adjusting the intensity of incentives to maximize the “Porter Effect” while reducing the short-term cost burden on enterprises, thereby helping them address practical challenges.

Secondly, in practice, the LCCP has contributed to the GTFP of enterprises [104]. Furthermore, from 2015 to 2020, the growth rate of green patent applications by Chinese listed companies surged by 1212.85% [106]. The LCCP should focus on improving the green input-output efficiency of enterprises, supporting green innovation, and institutionalizing green management.

Finally, given the time lag in policy implementation and the gradual emergence of policy effects, the government should strengthen coordination and information sharing across different policy years. Particularly for enterprises that joined the LCCP early, the government should provide more experience sharing, training, and policy guidance to help enterprises quickly adapt and achieve green innovation. For enterprises joining later, the government can offer more specific and flexible support measures based on the experiences of earlier pilot cities, thereby reducing the uncertainties faced by enterprises.

However, it is important to acknowledge the potential limitations of this study. The analysis relies on data from Chinese A-share listed companies, which raises concerns about the sample’s representativeness and whether the time span of the data is adequate to observe the long-term effects of policies. Moreover, including data from non-listed small and medium-sized enterprises could improve the generalizability of the research findings. These aspects warrant further examination in future studies.

## List of abbreviations:

LCCP: Low Carbon City Policy
GTFP: Green Total Factor Productivity
